# Molecular Surveillance Reveals Diverse Tick-Borne Pathogens in Northern California

**DOI:** 10.3390/pathogens15080855

**Published:** 2026-08-17

**Authors:** Alisa Rose Aboshi, Raeleen Valencia, Brandon A. Gomes, Arvind Sharma, Claudia Rückert, Mike Teglas, Andrew B. Nuss, Daniel Sarna-Wojcicki, Ben J. Saxon, Emilio Tripp, Jessica Camarena, Brandon Tripp, Monika Gulia-Nuss

**Affiliations:** 1 Department of Biochemistry, Molecular Biology & Biotechnology, University of Nevada, Reno (UNR), Reno, NV 89557, USA; aaboshi@unr.edu (A.R.A.); raeleenv@unr.edu (R.V.); bgomes@unr.edu (B.A.G.); crueckert@unr.edu (C.R.); 2 Department of Agriculture, Veterinary & Rangeland Sciences, University of Nevada, Reno, NV 89557, USA; nussab@unr.edu (A.B.N.); 3 Karuk Wildlife Team, Karuk Tribe Department of Natural Resources, Orleans, CA 95556, USA

**Keywords:** *Ixodes pacificus*, *Dermacentor similis*, *Dermacentor andersoni*, *Dermacentor occidentalis*, *Anaplasma phagocytophilum*, *Borrelia*, *Rickettsia*

## Abstract

Ticks are vectors of bacterial, viral, and protozoan pathogens and represent a growing public health concern in the United States and worldwide. In California, vector surveillance has largely focused on *Ixodes pacificus*, the major vector of the spirochete, *Borrelia burgdorferi*. However, the presence of multiple medically important *Dermacentor* species may harbor additional, understudied pathogens. To address this gap, broad pathogen screening was conducted on ticks collected from Siskiyou, Humboldt, Trinity, and Mendocino counties in Northern California, using multiplex quantitative PCR targeting 15 known tick-borne pathogens in the USA. A total of 376 adult ticks were collected in total, comprising *I. pacificus*, *D. similis*, *D. andersoni*, and *D. occidentalis*, from vegetation and animals between 2023 and 2024. Five established bacterial pathogens, *Anaplasma phagocytophilum*, *B. burgdorferi*, *B. miyamotoi*, *Rickettsia monacensis*, and *R. rhipicephali* were detected. Additionally, a genetically divergent *Rickettsia*-like sequence was detected in one *I. pacificus* sample that did not match any previously described species in phylogenetic analyses. Furthermore, a short DNA fragment highly similar to the Colorado tick-fever-virus VP12 gene was detected in *D. similis*. These findings suggest pathogen diversity across multiple tick species in Northern California, including evidence of co-infections, and the detection of potentially novel or poorly characterized microorganisms.

## 1. Introduction

Ticks are ectoparasites and obligate blood feeders during all life stages. Due to their dependence on vertebrate blood meals, they serve as significant vectors that transmit a range of pathogens, including bacteria, viruses, and protozoa [1]. In the United States, tick-borne diseases are an increasing public health threat, accounting for 95% of reported vector-borne infections [2,3]. *Ixodes pacificus* is one of the most significant arthropod vectors in western North America and transmits a wide range of pathogens, including species of *Anaplasma*, *Babesia*, *Bartonella*, *Borrelia*, *Ehrlichia*, and *Rickettsia* [4]. While *I. pacificus* distribution extends from British Columbia and Yukon in the north to Mexico in the south [5,6,7,8], a recent report identified *I. pacificus* on animals in Anchorage, Alaska [6,9], suggesting a potential range expansion of this tick species. While *I. pacificus* has a broad geographic range, most work on tick surveillance and ecology for this species has been conducted in California [10,11,12,13,14].

In addition to *I. pacificus*, 49 tick species spanning six genera, including two soft-tick genera (*Argas*, *Ornithodoros*) and four hard-tick genera (*Dermacentor*, *Haemaphysalis*, *Ixodes*, and *Rhipicephalus*) have been reported in California [15]. The majority of these ticks feed on wildlife and are strictly animal pests [1,15], but species such as *D. similis* (Western American dog tick, previously known as *D. variabilis*) and *D. andersoni* will feed on humans and are known vectors of *R. rickettsii*, which causes Rocky Mountain spotted fever, and *D. occidentalis* is a vector of *R. philipii*, which causes Pacific Coast tick fever [1,16]. In California, the incidence of tick-borne diseases in humans varies by pathogen: Lyme disease, caused by *B. burgdorferi*, is the most commonly reported, while anaplasmosis, babesiosis, and other tick-borne infections occur less frequently [17]. However, reported human incidence likely underestimates the true burden of disease because many infections potentially go undiagnosed or unreported [18]. Studies on pathogen surveillance in *D. variabilis* in the eastern United States have identified a diverse range of pathogens, including *Anaplasma*, *Rickettsia*, *Francisella*, and Powassan virus Lineage I [19,20,21,22,23]. Therefore, current surveillance efforts focusing solely on *I. pacificus* potentially overlook a broader pathogen diversity and tick–pathogen associations [24].

In this study, we address this gap by collecting multiple tick species from California and screening for a broad panel of known tick-borne pathogens in North America [25,26,27,28,29,30,31,32,33,34,35]. This broad-spectrum approach was designed to capture both pathogens previously documented in California and those with more geographically restricted distributions, thereby enabling surveillance for pathogens that may be emerging, expanding their geographic range, or otherwise underrecognized in this region. Using multiple collection methods (tick drags, removal from humans, companion animals, hunter-killed animals, or roadkill), we collected questing or feeding adult ticks during two seasons, May 2023 to June 2024, from Siskiyou, Humboldt, Trinity, and Mendocino counties in California. *Ixodes pacificus* was the most abundantly collected tick species, followed closely by *D. occidentalis*, whereas *D. similis* and *D. andersoni* made up a smaller portion of the total count. Of the 15 pathogens tested, five were identified in our tick samples. Additionally, we report a higher diversity of Rickettsial species than previously noted. These data support the need for better tick-borne pathogen surveillance throughout the region.

## 2. Materials and Methods

### 2.1. Tick Collection

**Figure 1:**
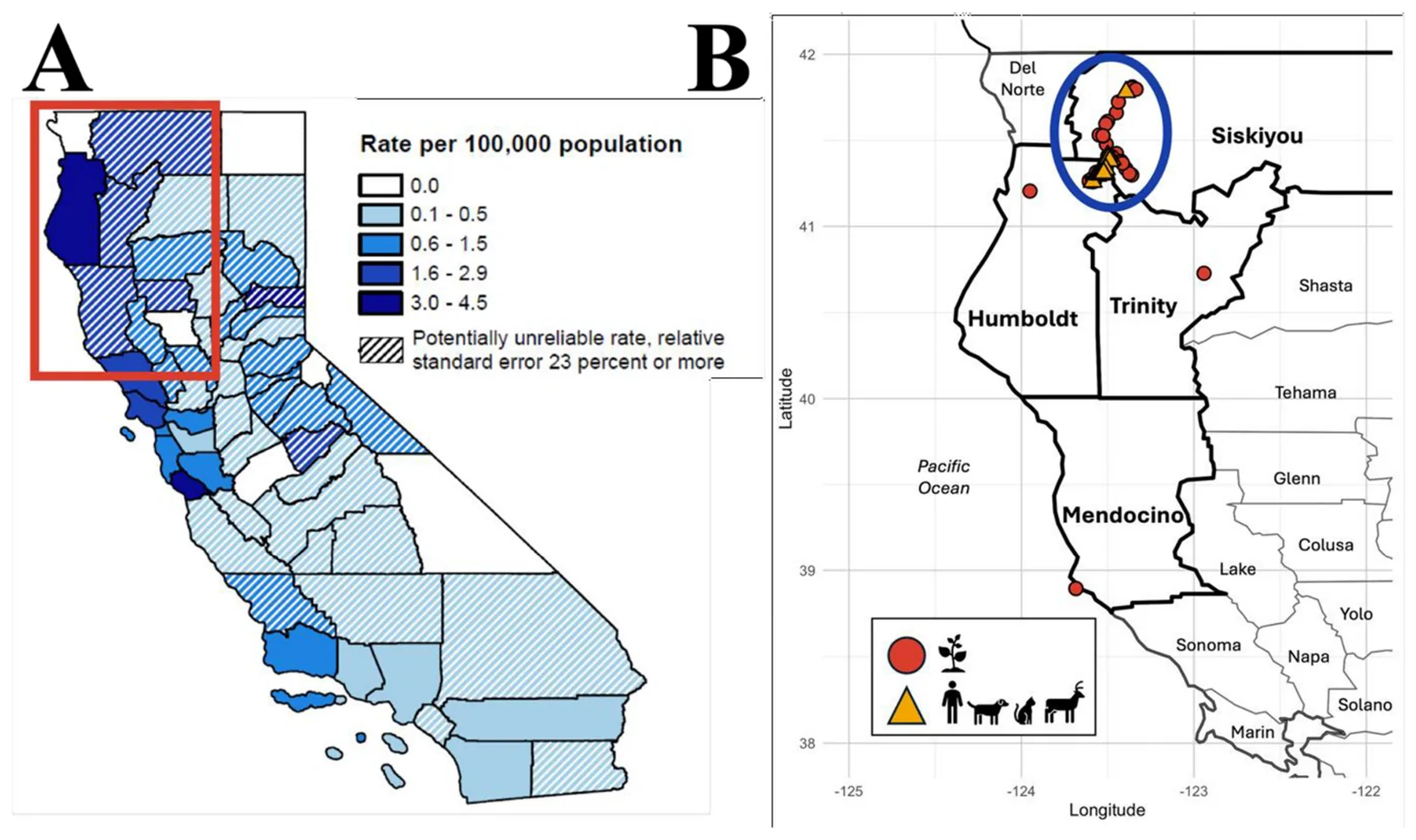
Maps showing Lyme disease incidence and the tick collection sites in California. (**A**) Average annual incidence rates of Lyme disease by county in California, 2013–2019. Figure adapted from the Epidemiologic Summary of Lyme Disease in California, 2013–2019, California Department of Public Health https://www.cdph.ca.gov/Programs/CID/DCDC/CDPH%20Document%20Library/LymeDiseaseEpiSummary2013-2019.pdf accessed on 4 February 2026. Counties where ticks were collected in the current study are indicated by the red square. (**B**) Tick collection sites in Siskiyou, Humboldt, Trinity, and Mendocino counties, California, USA. Ticks were collected from vegetation, humans, dogs, cats, and roadkill deer. Collection sites are indicated by red circles for ticks collected from vegetation and orange triangles for ticks collected from humans, dogs, cats, or roadkill deer. The Karuk Reservation, which extends across portions of Siskiyou and Humboldt counties, is indicated by a blue oval.

We focused on areas in Northern California with high rates of reported Lyme disease cases (Figure 1A) and because of our collaboration with the Karuk Tribe, the second largest Federally-recognized Native American tribe in California, with ancestral lands along the Klamath River in the state’s northwest (Figure 1B). As ticks are indicators of ecosystem health, signaling the complex interactions among wildlife, environmental conditions, and human exposure to infectious disease, the Karuk Wildlife Team initiated a pilot tick-borne disease sampling effort to evaluate and refine methods for monitoring ticks and associated pathogens within Karuk Aboriginal Territory. Ticks were collected between May, June and December of 2023 and monthly from January to June in 2024 either by the Karuk Tribe Department of Natural Resources team (Siskiyou and Humboldt counties) or the University of Nevada, Reno team (Humboldt, Trinity, and Mendocino counties). Ticks were collected either by dragging a canvas cloth across vegetation or by removal from humans, companion animals (including dogs and cats), or from hunter-killed or roadkill deer (Figure 1B and Table 1). All ticks were adults and had not yet started feeding when collected from animals (flat ticks). Additionally, metadata for each collection, including location coordinates, collection date, and time, was recorded. Ticks were identified to the species level based on morphological characteristics and stored at −20 °C until further analysis [15,36,37].

### 2.2. DNA Extraction

Ticks were individually homogenized using an autoclaved pestle in a 1.7 mL microfuge tube after freezing in liquid nitrogen. DNA was extracted by the phenol/chloroform method. In brief, 700 μL of the extraction buffer (500 mM Tris, pH 9.0, 20 mM EDTA, 10 mM NaCl), 50 μL of 20% SDS, and 50 μL of Proteinase K (10 mg/mL) were added to the homogenized sample and incubated overnight (<16 h) at 55 °C. The incubated samples were centrifuged to remove debris, then treated with 10 μL of RNase A (20 mg/mL) at room temperature for 10 min, followed by incubation at 70 °C for 10 min to denature the enzyme. Each tube received 800 µL of Phenol:Chloroform:Isoamyl alcohol (Invitrogen, Waltham, MA, USA), vortexed thoroughly and centrifuged at 13,300 rpm for 15 min. The upper aqueous phase (~600 µL) was collected and transferred to a fresh 1.7 mL tube, and 800 µL of chloroform was added. Chloroform extraction was conducted twice, and the aqueous phase was collected. A total of 3 M sodium acetate, pH 5.2, was added to the aqueous phase (1/10 of total volume, ~20 µL), mixed by flicking the tube, and precipitated with 1 volume (aqueous phase) of 95% ethanol and kept at −20 °C overnight. Samples were centrifuged at 13,300 rpm for 25 min, washed twice with 1 mL of 70% ethanol, and centrifuged at 13,300 rpm for 10 min. Finally, the washed DNA pellets were air-dried, resuspended in 25 µL of nuclease-free water, and incubated at 55 °C for 20 min. The extracted DNA was placed onto 1.5% agarose gel to assess DNA quality and quantified using a NanoDrop One (Thermo Fisher Scientific, Waltham, MA, USA).

### 2.3. Dermacentor Similis Validation from Collected Ticks

To validate *D. similis*, previously classified as *D. variabilis*, we conducted PCR amplification, sequencing, and phylogenetic analysis of the cytochrome c oxidase subunit I (COI) gene following morphological identification as used in a previous study [38]. PCR was conducted using Apex Taq RED Master Mix (Genesee Scientific, El Cajon, CA, USA) to amplify a 709 bp region of the COI gene under amplification conditions used in previous studies [39]. Amplicons were sequenced in both directions and aligned using Clustal Omega v. 1.2.4 on SnapGene v. 8.2.2 (SnapGene, MA, USA). Forward and reverse reads were manually inspected, trimmed to remove low-quality bases, and assembled into consensus sequences. Multiple alignments were conducted using SnapGene v. 8.2.2 with the Crustal Omega model, along with previously reported COI sequences from *D. similis* and *D. variabilis* available from the NCBI database. Subsequently, a phylogenetic dendrogram was constructed using the maximum likelihood method in MEGA X using the maximum likelihood method with the Tamura 3-parameter model (1992) + G, and the bootstrap support was assessed with 1000 replicates [40,41].

### 2.4. Multiplex Quantitative Polymerase Chain Reaction for Tick-Borne Pathogen Screening

A multiplex quantitative polymerase chain reaction (qPCR) was used to detect known tick-borne pathogens in North America (Table 2). Primer and probe sequences for *R. rickettsii* [29] and *B. miyamotoi* [26] were obtained from published work. Primer and probe sequences for the other 13 known tick-borne pathogens were obtained from Dr. Saravanan Thangamani (State University of NY, Upstate Medical University, Syracuse, NY). Each qPCR was conducted with primer groups Mix A, Mix B, Mix C, Mix D, Mix E, and Mix F (Table 2).

qPCR was performed on a CFX96 Touch Real-Time PCR Detection System (Bio-Rad Laboratories, Irvine, CA, USA) using a qPCRBIO Probe Mix Lo-ROX (PCR Biosystems, London, UK) following the manufacturer’s protocol. For the qPCR, nuclease-free water was used for a non-template control, and primers targeting the tick ribosomal S4 gene were used as a housekeeping control [42]. Positive controls for *A. phagocytophilum*, *B. microti*, and *E. chaffeensis* were obtained through BEI Resources, NIAID, NIH: strain NCH-1, NR-51150; strain GI, NR-50774; and strain Liberty, NR-50091, respectively. For other pathogens, a synthetic DNA fragment corresponding to the targeted sequence was procured and used as a positive control (Twist Bioscience, San Francisco, CA, USA). The amplification temperature consisted of an initial denaturation at 95 °C for 10 min, followed by 40 cycles of 95 °C for 11 s, and 60 °C for 30 s. A final melt curve analysis was conducted from 65 °C to 95 °C. Positive DNA samples were identified by a quantification cycle (Cq) value < 30, similar to other published work [26,29].

### 2.5. Rickettsia spp. Identification and Colorado Tick-Fever-Virus Validation

Due to the similarity among *Rickettsia* species and the short qPCR primer lengths (<150 bp), we conducted additional PCR screening and sequencing of *Rickettsia*-positive samples. qPCR *Rickettsia*-positive samples were subjected to PCR using Apex Taq RED Master Mix (Genesee Scientific, El Cajon, CA, USA) targeting 632 bp of the outer-membrane protein A (*ompA*), 1022 bp of the outer-membrane protein B (*ompB*), and 1156 bp of the citrate synthase (*gltA*) genes, using the amplification conditions used in previous studies [43,44] (Table 3). The amplified PCR products were confirmed by agarose gel electrophoresis, purified with the MinElute Gel Extraction Kit (Qiagen, Hilden, Germany), and sent for Sanger sequencing to the Nevada Genomics Center (University of Nevada, Reno, NV, USA).

One sample that tested positive for Colorado tick fever virus (CTFV) by multiplex qPCR was further validated using a single plex conventional PCR assay with the same VP12-targeting primer set (114 bp). The PCR product was confirmed by agarose gel electrophoresis and purified with the MinElute Gel Extraction Kit (Qiagen) and sequenced with the Sanger sequencing platform at the Nevada Genomics Center (Table 3). Sequences were then identified using BLAST searches on the NCBI database (NCBI BLAST+). Multiple alignments were performed using Clustal Omega v. 1.2.4 on SnapGene v. 8.2.2 with previously reported CTFV VP12 sequences on GenBank.

### 2.6. Phylogenetic Data Analysis

Amplicons of the expected sizes were sequenced in both directions and aligned using Clustal Omega v. 1.2.4 on SnapGene v. 8.2.2 (SnapGene) and assembled as described previously. The resulting sequences were queried against the NCBI database using BLAST analysis. The newly generated nucleotide sequences were deposited in the International Nucleotide Sequence Database (GenBank) under accession numbers PZ439541-PZ439543. Phylogenetic analyses of *Rickettsia*-positive samples were conducted using the partial *ompA*, *ompB*, and *gltA* genes. Multiple alignments were performed using Clustal Omega v. 1.2.4 on SnapGene v. 8.2.2, and alignments were manually curated to remove poorly aligned regions and gaps at the ends of sequences prior to phylogenetic analysis. Phylogenetic trees were constructed in MEGA X using the maximum likelihood method with the Tamura 3-parameter model (1992) + G + I and the bootstrap support was assessed with 1000 replicates [40,41].

## 3. Results

### 3.1. Tick and Pathogen Diversity

A total of 376 adult ticks, comprising *I. pacificus* (154 total), *D. occidentalis* (128 total), *D. similis* (67 total), and *D. andersoni* (27 total), were collected from Siskiyou, Humboldt, Trinity, and Mendocino counties (Table 1). *Ixodes pacificus* was collected from all four counties, whereas *D. similis*, *D. andersoni*, and *D. occidentalis* were collected only in Siskiyou and Humboldt counties. Phylogenetic analysis of the COI gene in *D. variabilis* and *D. similis* confirmed that the *D. similis* collected in this study formed a clade with previously reported *D. similis* or *D. variabilis* collected in western United States (now *D. similis*) (Figure S1). This clade was separate from *D. variabilis* collected in eastern Canada and the United States, indicating that the *D. similis* collected in this study is different from *D. variabilis*, as previously reported in California [45,46,47] (Figure S1). Molecular genetic screening detected the presence of *A. phagocytophilum*, *B. burgdorferi*, *B. miyamotoi*, *Rickettsia* species, and a CTFV-like sequence. None of the ticks tested positive for the remaining pathogens included in the screening (Table 2).

### 3.2. Detection of Anaplasma phagocytophilum, Borrelia burgdorferi, and Borrelia miyamotoi

*Anaplasma phagocytophilum*, *B. burgdorferi*, and *B. miyamotoi* were each detected in 1.95% (3/154) of *I. pacificus* collected in Siskiyou and Humboldt counties combined (Table 1). Molecular screenings for *A. phagocytophilum* and *B. burgdorferi* were only conducted on *I. pacificus*, whereas all collected tick species were screened for *B. miyamotoi*. Siskiyou County had the highest diversity of detected pathogens in *I. pacificus*, with all three pathogens present. Additionally, one *I. pacificus* tick in Siskiyou County tested positive for both *A. phagocytophilum* and *B. burgdorferi* indicating co-infection (Table 1).

### 3.3. Detection of Rickettsia spp. in Ixodes and Dermacentor Ticks

**Figure 2:**
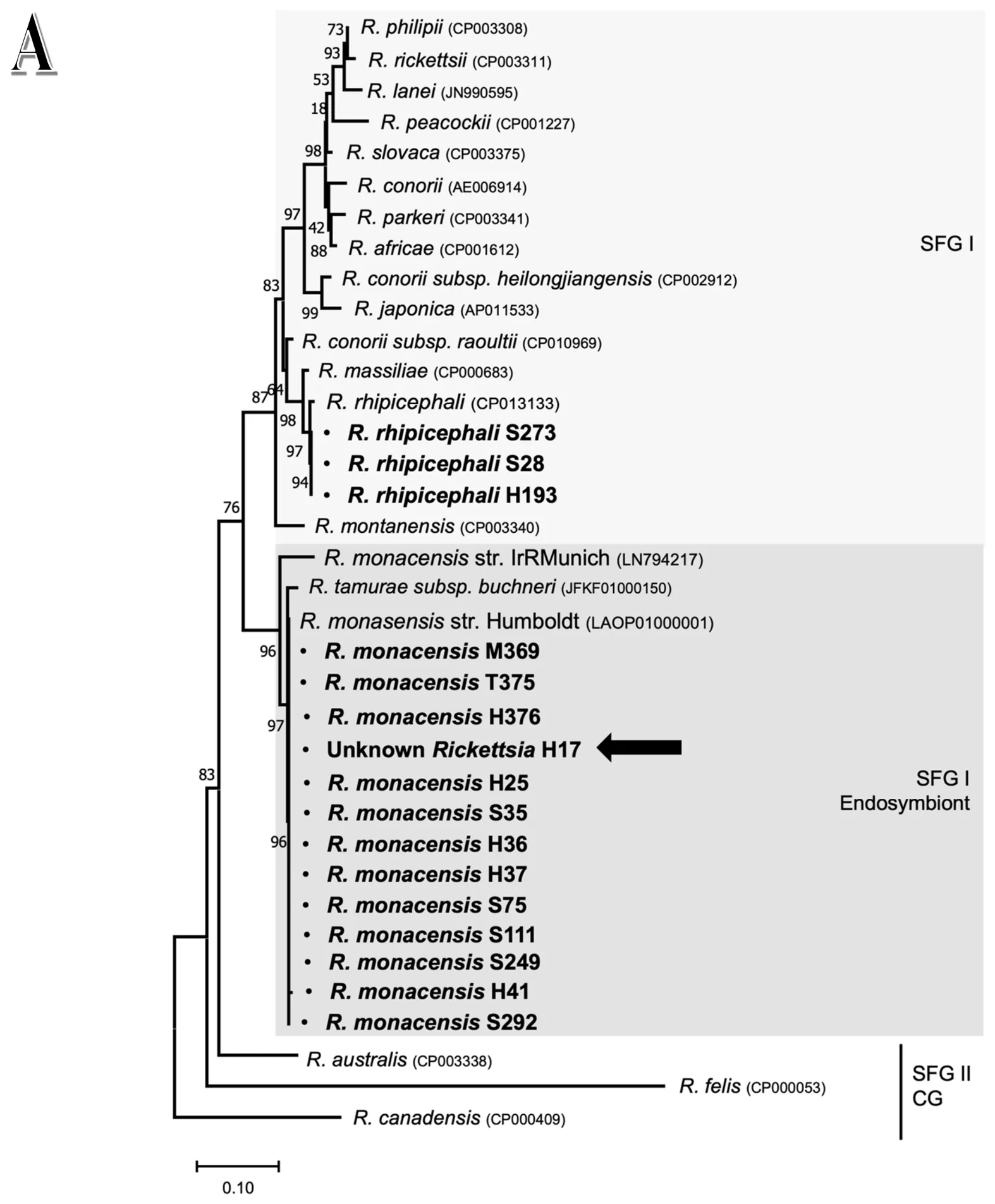

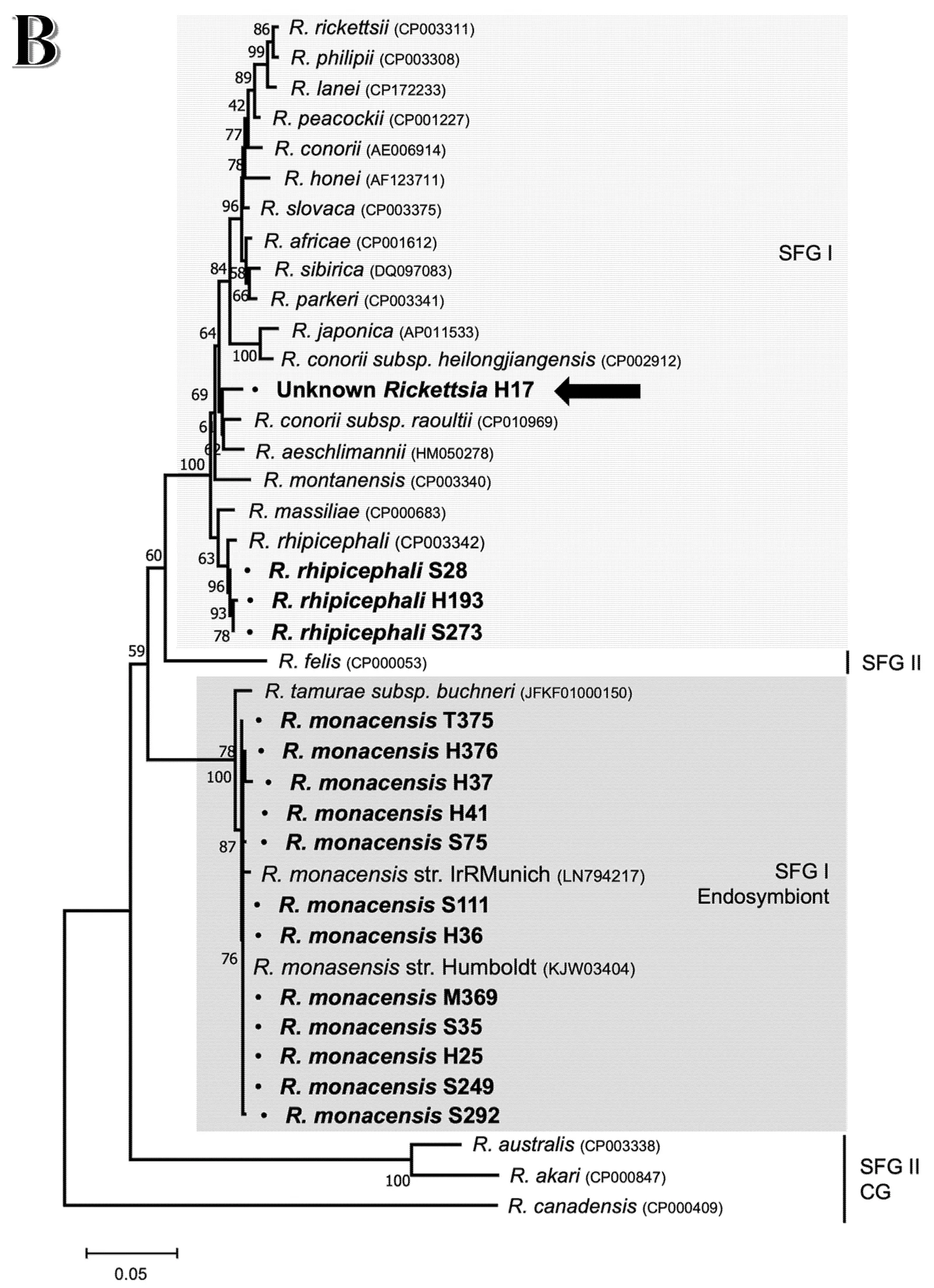

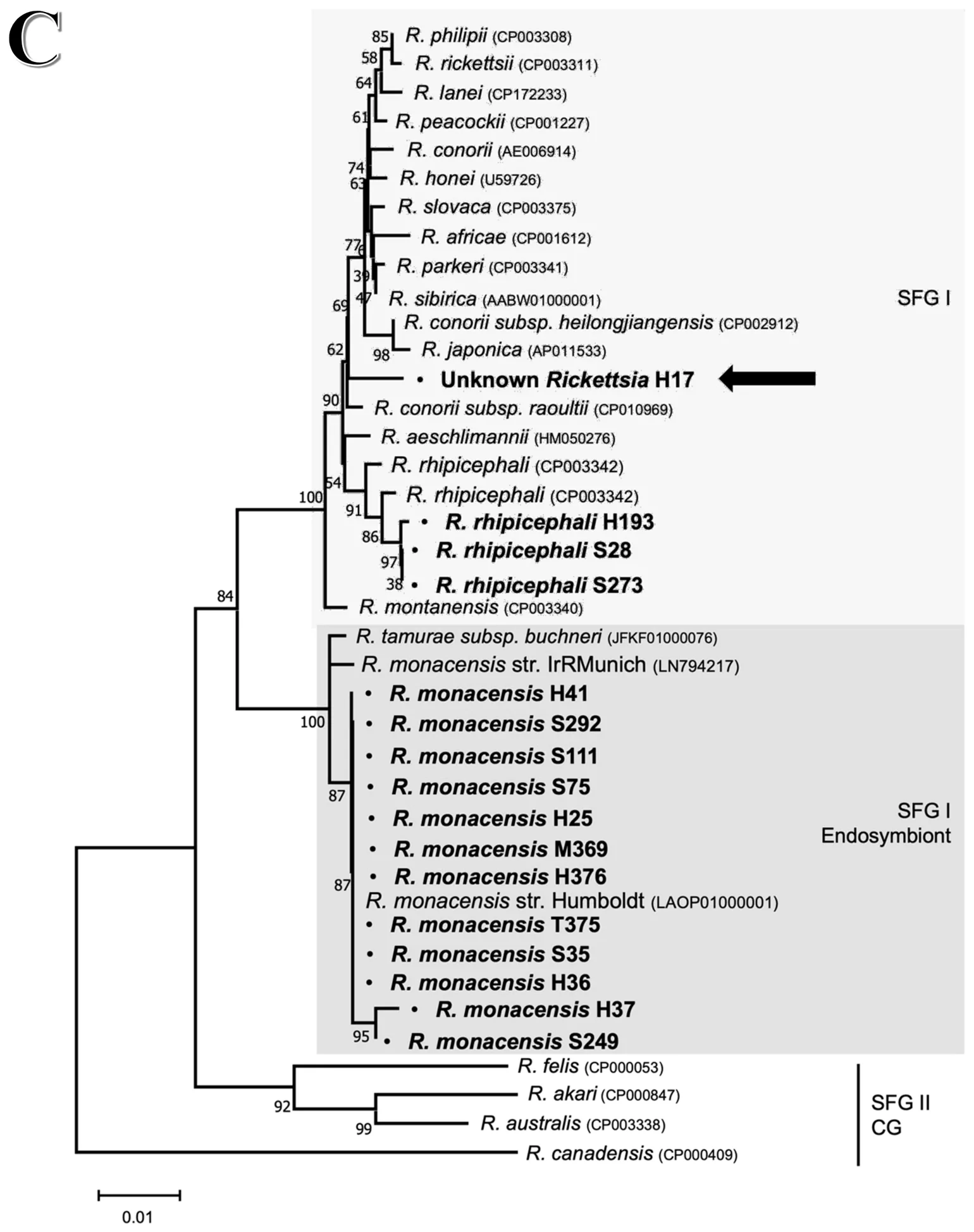
Phylogenetic analysis of Rickettsia species. Phylogenetic tree based on partial sequences of the (**A**) *ompA*, (**B**) *ompB*, and (**C**) *gltA* genes of *Rickettsia* amplified from tick samples collected in this study. Sequences generated in this study are shown in bold text, with the sample number and the corresponding collection counties: S, Siskiyou; H, Humboldt; T, Trinity; M, Mendocino. Additionally, sample H17 is indicated with a black arrow. Major phylogenetic groupings within the genus *Rickettsia* are indicated, including spotted-fever group I (SFGI), spotted-fever group II (SFGII), and the ancestral group (CG) based on [49]. Reference sequences of representative spotted-fever group *Rickettsia* were obtained from GenBank (accession numbers listed in parentheses and Table S1). The tree was constructed using the maximum likelihood method and the Tamura 3-parameter model (1992) + G + I in MEGA X with bootstrap values (1000 replicates) shown at the nodes. The full length obtained from each representative strain was used in this study (*ompA*; ~514 bp, *ompB*; 2176 bp, *gltA*; 1087 bp).

*Rickettsia ompA*, *ompB*, and *gltA* genes were successfully amplified from 119 *I. pacificus*, two *D. occidentalis*, and one *D. andersoni* species. The *ompA*, *ompB*, and *gltA* genes are frequently targeted in *Rickettsia* identification [43,44]. The most common *Rickettsia* species detected in *I. pacificus* was *R. monacensis*, with an infection rate of 76.62% (118/154), and was detected in ticks collected from all four counties (Table 1). From the *R. monacensis*-positive samples, we selected five representative samples each from Siskiyou and Humboldt counties, and one each from Mendocino and Trinity counties, to construct a phylogenetic tree with other known spotted-fever group *Rickettsia* species using the *ompA*, *ompB*, and *gltA* genes (Figure 2A–C) (Table S1). All genes showed that the representative samples formed a clade with previously reported endosymbiotic *Rickettsia* species. Moreover, the *ompA* and *gltA* genes indicated that the representative samples clustered with *R. monacensis*, specifically the Humboldt strain (also known as *Rickettsia* species phenotype G021), previously detected in *I. pacificus* from Humboldt County, California [48] (Figure 2A,C).

None of the genes, *ompA* (514 bp), *ompB* (947 bp), and *gltA* (1087 bp) from the *I. pacificus* sample H17, showed 100% nucleotide sequence identity to the currently described *Rickettsia* species in GenBank. The *ompA* sequence shared 99.81% identity with the *I. pacificus* endosymbiont outer-membrane protein A-like gene (GenBank: GU047354), the *ompB* sequence (947 bp) shared 97.09% identity with *Candidatus Rickettsia tasmanensis* strain T152 outer-membrane protein B gene (GenBank: GQ223393), and the *gltA* sequence (1087 bp) shared 99.36% identity with the *Rickettsia* sp. strain YN02 citrate synthase gene (GenBank: KY411135.1) [50,51,52]. To further characterize this microorganism, we performed an additional PCR targeting the *ompB* gene to obtain a longer amplicon (2176 bp) for improved phylogenetic resolution (Table 2). Phylogenetic analyses were performed using *ompA*, *ompB* (2176 bp), and *gltA* gene sequences, which clustered it within the spotted-fever group *Rickettsia* (SFGR) lineage (Figure 2A–C). The *ompA* gene was grouped within the SFGR I endosymbiont clade (Figure 2A). For the *ompB* and *gltA* genes (Figure 2B,C), H17 appeared distinct from the primary *R. monacensis* clusters.

Phylogenetic analysis of the *Rickettsia* genes amplified from *D. occidentalis* and *D. andersoni* clustered consistently with *R. rhipicephali* across three gene trees (Figure 2A–C). *R. rhipicephali* was detected in 1.56% (2/128) and 3.70% (1/27) of *D. occidentalis* and *D. andersoni,* respectively. The *ompA* gene had the strongest bootstrap support of 98% (Figure 2A), followed by the *ompB* gene (96% bootstrap values) (Figure 2B), and the *gltA* gene supported clustering with *R. rhipicephali* with an 86% bootstrap value (Figure 2C).

### 3.4. Colorado Tick-Fever-Virus-like Sequence Detected in qPCR and Sanger Sequencing

One *D. similis* DNA sample tested positive for CTFV by qPCR. To confirm this result, we performed an additional conventional PCR with the same primer set as the qPCR assay, followed by Sanger sequencing of the amplicon. A 96 bp sequence was obtained, and a BLAST search resulted in only one hit that showed 100% nucleotide identity to the CTFV VP12 gene region. Additionally, multiple sequence alignments with previously reported CTFV VP12 sequences and CTFV VP12-like sequences showed high homology among the CTFV-like sequences detected in this study (Figure S2). To further assess the presence of DNA derived from the entire viral segment, we performed additional PCR targeting the near-complete VP12 region, using the complete sequence of the CTFV Florio strain (GenBank: NC_004190) [53], with synthesized target DNA as the positive control. However, no amplification was obtained. Therefore, only a 96 bp fragment corresponding to approximately 675 bp of the VP12 segment was successfully recovered from the *D. similis* in this study.

## 4. Discussion

In this study, we collected four tick species from vegetation, humans, companion animals, and deer. In our collections, *I. pacificus* was the most abundant (41%), followed closely by *D. occidentalis* (34%), whereas *D. similis* (18%) and *D. andersoni* (7%) made up a smaller portion of the total count (Table 1). Previous surveillance studies that include multiple tick species have shown that *I. pacificus* and *D. occidentalis* are generally reported as the most abundant in California [12,54,55,56,57,58,59]. However, seasonal variation influences these patterns, with other *Dermacentor* species found more frequently in the field in the fall [60]. Our collections occurred in winter (December–February) and spring (March–June) when adult ticks are more abundant. The nymphs commonly found in summer (June–August) in Northern California, therefore, were not present in our collection. Future surveillance incorporating the sampling of adult, nymphal, and larval stages across multiple seasons would provide a more comprehensive assessment of tick community composition and pathogen prevalence in Northern California.

Five pathogens, including *A. phagocytophilum*, *B. burgdorferi*, *B. miyamotoi*, *R. monacensis*, and *R. rhipicephali*, were detected in this study using qPCR and PCR, all of which have been previously reported in ticks from California [4,11,14,46,47,55,60,61,62,63]. Siskiyou County, which yielded the highest number of *I. pacificus* collections, also exhibited the greatest pathogen diversity (*A. phagocytophilum*, *B. burgdorferi*, *B. miyamotoi*, and *R. monacensis*), followed by Humboldt (*A. phagocytophilum*, *B. miyamotoi*, and *R. monacensis*), Mendocino, and Trinity counties (*R. monacensis*) (Table 3). The overall infection rate of all three pathogens (*A. phagocytophilum*, *B. burgdorferi*, and *B. miyamotoi)* was 1.95% each (3/154). These findings are consistent with previous reports in California, where *A. phagocytophilum* prevalence in adult *I. pacificus* ranged from approximately 0.3% to 7.8%, *B. burgdorferi* was between ~0.9 and 4%, and *B. miyamotoi* typically ranged from ~0.4 to 2%, with some areas reporting as high as ~3.9% [11,12,13,14,45,64]. Furthermore, the absence of these pathogens in counties where we did not detect them may not necessarily indicate their absence, but rather limited detection due to smaller sample sizes. Standardizing collection efforts across regions will be important for accurately comparing pathogen prevalence and distribution in future surveillance studies. Notably, we detected both *A. phagocytophilum* and *B. burgdorferi* in one *I. pacificus* sample from Siskiyou County, representing the first report of co-infection in this county. *Ixodes pacificus* co-infected with *A. phagocytophilum* and *B. burgdorferi* has been documented in Santa Cruz County, CA, although such occurrences remain rare [45]. Additionally, recent surveillance data from the California Department of Public Health indicate that co-infections with *A. phagocytophilum* and *B. burgdorferi* in *I. pacificus* are rare statewide, with only sporadic detections reported [17].

Members of *Rickettsia* are Gram-negative, obligate intracellular bacteria with diverse ecological roles and pathogenicity and are classified into ancestral, typhus, transitional, and SFGR groups I and II, reflecting their evolutionary relationships and host associations [49,65,66]. In this study, *R. monacensis* was detected in *I. pacificus* from all sampled counties, with consistently high infection rates ranging from 74.51% to 100% (Table 3). *Rickettsia monacensis* was first identified in *I. ricinus* ticks in Europe and has been considered an endosymbiotic member of the SFGR [67]. Although there have been no human infections in the United States, rising reports of human infections in Europe underline its status as an emerging pathogen [68,69,70,71]. Previous studies have also documented *R. monacensis* str. Humboldt in *I. pacificus* from California, including Humboldt and Napa counties, supporting its established presence in the region [48,63]. The high prevalence observed in this study is consistent with other reports of widespread *R. monacensis* infection in tick populations [72,73,74]. This may be partly explained by efficient transovarial transmission, documented in *R. monacensis* str. Humboldt, as well as among other SFGR groups [75,76,77]. Overall, detection in all four counties and the high prevalence of *R. monacensis* in *I. pacificus* suggest that this *Rickettsia* species is well established throughout Northern California.

Furthermore, we detected a genetically distinct *Rickettsia*-like sequence in one *I. pacificus* sample (termed H17). Although the amplification of the *ompA*, *ompB*, and *gltA* genes confirmed its placement within the SFGR clade, this organism did not show complete sequence identity with any currently described *Rickettsia* species. BLAST analysis revealed similarity to multiple reference sequences, including an *I. pacificus* endosymbiont for the *ompA* gene (99.81% nucleotide similarity), *Candidatus Rickettsia tasmanensis* for *the ompB* gene (97.79%), and another uncharacterized *Rickettsia* strain for the *gltA* gene (99.36%) [50,51,52]. Phylogenetic analyses consistently placed sample H17 within the SFGR lineage across all three genes. The placement of H17 within the SFGR I endosymbiont clade based on *ompA* suggests a closer relationship with endosymbiotic lineages (Figure 2A). Similarly, the distinct placement of H17 relative to the *R. monacensis* and *R. rhipicephali* clusters in the *ompB* and *gltA* genes may reflect a genetic divergence (Figure 2B,C). Overall, the inconsistent placement across genes suggests that the genetic markers used do not provide sufficient phylogenetic resolution to confidently determine their evolutionary relationships. The lack of consistent placement across genes suggests that this *Rickettsia*-like sequence may represent a genetically divergent lineage. There are specific criteria for establishing a novel *Rickettsia* species, including the isolation of the bacterium and the isolate falling below defined nucleotide identity thresholds across multiple genes (*ompA*, *ompB*, *gltA*, and *16S RNA*) and forming a distinct phylogenetic lineage within each gene [78]. While our sample showed divergence from known species and inconsistent clustering across the *ompA*, *ompB*, and *gltA* genes, the current results are insufficient to definitively classify it, as we did not attempt to culture the microorganism. Additional analyses, including bacterial culture or whole-genome sequencing, are required to clarify its taxonomic identity and assess its potential as an SFGR pathogen.

Several *Dermacentor* tick species of public health importance, including *D. similis*, *D. andersoni*, and *D. occidentalis*, were also collected. *Dermacentor similis* is a newly described tick species found in Western North America, formerly recognized as *D. variabilis* [37]. The California Department of Public Health suggests that while the predominant species in California is *D. similis*, there may be areas where *D. variabilis* occurs [79]. Therefore, we conducted a genomic analysis and confirmed that the ticks collected in this study were *D. similis*. *Dermacentor similis* is a well-established vector of *R. rickettsii* in California [80], however *R. rickettsii* or any other *Rickettsia* species was not detected in *D. similis* ticks collected in this study. Instead, we detected a 96 bp sequence similar to the VP12 region of CTFV in *D. similis* collected from Siskiyou County. CTFV is a segmented, double-stranded RNA virus primarily transmitted by *D. andersoni* ticks in the Rocky Mountain region of the United States and Canada [81,82]. CTFV is a historical tick-borne pathogen in the United States, with the first recognition of CTF in the 1850s and studies of CTFV and ticks dating to the mid-20th century. Reported infection rates in *D. andersoni* have varied by region and time, from 8 to 40% in early studies, to ~4% more recently in California, 21% in Wyoming, and 58% in Colorado [62,83,84,85,86,87,88]. Although the main vector of CTFV is *D. andersoni*, previous studies show that CTFV can infect other *Dermacentor* species, including *D. occidentalis*, *D. albopictus*, *D. parumapterus*, and *D. arumapertus* [62,85,89]. Notably, CTFV has been detected in *D. occidentalis* ticks in Siskiyou County [62]; therefore, this possible evidence of prior infection in *D. similis* is not entirely unexpected and suggests a broader host range for CTFV than previously known. Since we detected only a CTFV-like DNA sequence, not RNA, it could be an endogenous viral element (EVE) [90] or virus-derived DNA post-infection [91]. Further investigation is needed to determine whether CTFV can infect *D. similis* in this region.

*Dermacentor andersoni* are vectors of *R. rickettsii*, and *D. occidentalis* are vectors of *R. philipii* and potentially for the newly recognized *R. lanei* in California [1,16,57]. However, these *Rickettsia* species were not detected in this study. We detected *R. rhipicephali* in *D. andersoni* and *D. occidentalis* in Siskiyou and Humboldt counties. *Rickettsia rhipicephali* is an SFGR, first identified from *R. sanguineus* ticks collected in southeastern United States [92]. Now, *R. rhipicephali* has been reported in ticks from multiple regions across western and southern United States [46,47,55,60,61,93,94,95,96,97,98,99]. Although *R. rhipicephali* is traditionally considered a non-pathogenic SFGR and human cases have not been reported, infection experiments on guinea pigs have shown mild pathogenicity [100]. *Rickettsia rhipicephali* has been previously reported in *D. andersoni* from Washington state [95]; however, this is the first report from California. The presence of *R. rhipicephali* from two *Dermacentor* species in the same geographic region may suggest a broader vector range, facilitating its maintenance and spread within enzootic cycles.

Our findings reveal greater complexity in tick-borne pathogen dynamics, including co-infections, high infection rates with *Rickettsia* species, and possible expansion of vectors for some pathogens. Although *I. pacificus* is the primary focus of most surveillance studies in California, our results demonstrate that *Dermacentor* ticks also harbor diverse pathogens and should not be overlooked. These findings highlight the limitations of narrowly targeted surveillance methods and emphasize the need for broader, multi-pathogen screening across various tick species. Additionally, these efforts represent an important step toward establishing a framework for engaging Tribal communities in monitoring tick-borne diseases in Tribal lands to protect wildlife and community health and strengthen long-term stewardship of Indigenous ecosystems.

## Figures and Tables

**Table 1:** Number of ticks collected from California counties and pathogen infection rates.

County	Species	Number of Ticks Collected		Infection Rate (# Positive/# Tested)					
		Vegetation	Animals	*Anaplasma phagocytophilum*	*Borrelia burgdorferi*	*Borrelia miyamotoi*	*Rickettsia monacensis*	*Rickettsia rhipicephali*	Colorado Tick Fever Virus
Siskiyou	*I. pacificus*	70	13	1.08% (1/93)	3.23% (3/93)	2.15% (2/93)	77.42% (72/93)		
	*D. similis*	50							2% (1/50)
	*D. andersoni*	19						5.26% (1/19)	
	*D. occidentalis*	104						0.96% (1/104)	
Humboldt	*I. pacificus*	34	17	3.92% (2/51)		1.96% (1/51)	74.51% (38/51)		
	*D. similis*	8	9						
	*D. andersoni*	8							
	*D. occidentalis*	20	4					4.17% (1/24)	
Mendocino	*I. pacificus*	9					77.78% (7/9)		
Trinity	*I. pacificus*	1					100% (1/1)		
Total	*I. pacificus*	124	30	1.95% (3/154)	1.95% (3/154)	1.95% (3/154)	76.62% (118/154)		
	*D. similis*	58	9						1.49% (1/67)
	*D. andersoni*	27						3.70% (1/27)	
	*D. occidentalis*	124	4					1.56% (2/128)	

Number (#) of samples tested positive in our assays out of total samples tested.

**Table 2:** List of pathogens and qPCR primer sets for pathogen detection.

Multiplex Groups ^a^	Target Pathogen	Target Gene	Sequence (5′–3′)			Product Length (bp)	Accession No.	Reference
			Forward	Reverse	Probe			
Mix A	*Borrelia burgdorferi*	*ospA*	AGTGCCTGAATTCCAAGCTG	CATTGGTGGTTAAAGAAGGAACTG	FAM-AGTAGCAGCACTACTGTCAGTGTCA-BHQ1	131	MK543500	[27]
	*Anaplasma ph-agocytophilum*	*msp2*	ATGGAAGGTAGTGTTGGTTATGGTATT	TTGGTCTTGAAGCGCTCGTA	JOEN-TGGTGCCAGGGTTGAGCTTGAGATTG-BHQ1	77	MH734193	[33]
Mix B	Powassan virus	NS5	CCGAGCCARRGTGAGGATGT	TCTTTTGCYGARCTCCACTT	Cy5-TTCATAGCGAAGGTKAGRTCCAACG-BHQ1	111	MK733761	[27]
	*Ehrlichia muris* subsp. *eauclairensis*	*p13*	AAAGAGTCAGTGTTGATCCG	CAATGATGATACTGCGAACA	TEX615-TTGCTATTGGCYGGGTTTTTTAGT-BHQ1	100	KF523727	[25]
	*Ehrlichia chaffeensis*	*dsb*	TATTGCTAATTACCCTCAAAAAGTC	GAGCTATCCTCAAGTTCAGATTT	HEX- ATTGACCTCCTAACTAGAGGGCAAGCA -BHQ2	117	AF403711	[32]
	Heartland virus	S segment	CCTTTGGTCCACATTGTTG	CACTGATTCCACAGGCAGAT	FAM-TGGATGCCTATTCCCTTTGGCAA-TAMTSp	107	JX005843	[34] ^b^
Mix C	*Babesia duncani*	*beta-tubulin*	CGGCATTGACCCTGTAA	CGGTGGCCTCATTGTAA	TEX615-TTGCTATACACCCCCCCGAATAA-BHQ2	124	MF978361	Designed ^c^
	*Bartonella henselae*	*pap13*	CCGCTGATCGCATTATGCCT	AGCGATTTCTGCATCATCTGCT	HEX-ATGTTGCTGGTGGTGTTTCCTATGCAC-BHQ1	107	AB091503	[32]
	*Babesia microti*	18s rRNA	GGGACTTTGCGTTCATAAAACGC	GCAATAATCTATCCCCATCACGAT	FAM-TTAGATGTCCTGGGCTGC-BHQ1	171	MK609547	[35] ^d^
Mix D	Colorado tick fever virus	VP12	TTCGGCAGTACGCAATGT	AAATCGGCGGGAGAATAA	Cy5-AAGACATCACCACTTGCCTCC-BHQ2	114	KY454401	Designed ^c^
	Tick-borne encephalitis virus	NS2A	TCAATCTGGAAACTGATTCGA	GAGGTGTGCCGCATC	HEX-GGAGCTGGCCATCCATG-BHQ1	212	JF819648	Designed ^c^
	*Rickettsia parkeri*	*ompB*	CAAATGTTGCAGTTCCTCTAAA	AAAACAAACCGTTAAAACTACCG	FAM-AATTAATACCCTTATGARCASCAGCAG-BHQ1	96	AF123717	[28] ^d^
Mix E	Bourbon virus	S segment	AACCGAAGGACCATTGCTAC	ACAGGGACTCCAGAACTTGG	FAM-ACCCTTGCTGCATCTTCCACCA-TAMSp	110	MK453528	[30] ^b^
	*Borrelia miyamotoi*	*purB*	TCCTCAATGATGAAAGCTTTA	GGATCAACTGTCTCTTTAATAAAG	TEX615-TCGACTTGCAATGATGCAAAACCT-BHQ2	121	CP004217	[26] ^b^
Mix F	*Rickettsia rickettsii*	*gltA*	GAGAGAAAATTATATATCCAAATGTTGAT	AGGGTCTTCGTGCATTTCTT	FAM- CATTGTGCCATCCAGCCTACGGT-BHQ1	147	CP130457	[29]

^a^ Each qPCR was conducted with primer groups Mix A, Mix B, Mix C, Mix D, Mix E, and Mix F. ^b^ Original primer modified for this study. ^c^ Primer/probe design provided by Dr. Saravanan Thangamani, Director, SUNY Center for Environmental Health and Medicine/Vector Biology Laboratories, NY, USA. ^d^ Probe design provided by Dr. Saravanan Thangamani, Director, SUNY Center for Environmental Health and Medicine/Vector Biology Laboratories, NY, USA.

**Table 3:** PCR parameters and amplicon sizes for qPCR validation used for *Rickettsia* spp. and Colorado tick-fever-virus sequence validation.

Target Pathogen	Target Gene	Primer Name	Sequence (5′–3′)	Product Length (bp)	Amplification Parameters	Reference
*Rickettsia* spp.	*ompA*	RR190-70	ATGGCGAATATTTCTCCAAAA	632	98 °C for 1 min, 45 cycles of 98 °C for 10 s, 60 °C for 20 s, 72 °C for 40 s, hold at 72 °C for 2 min	[43]
		RR190-701	GTTCCGTTAATGGCAGCATCT			
	*ompB*	120-1378	TAAACTTGCTGACGGTACAG	1022	95 °C for 3 min, 40 cycles of 95 °C for 30 s, 50 °C for 30 s, 72 °C for 30 s, hold at 72 °C for 5 min	[44]
		120-2399	CTTGTTTGTTTAATGTTACGGT			
		120-2113 ^a^	CGATGCTAACGTAGGTTCTT	876		
		120-2988 ^a^	CCGGCTATACCGCCTGTAGT			
		120-2788 ^a^	AAACAATAATCAAGGTACTGT	812		
		120-3599 ^a^	TACTTCCGGTTACAGCAAAGT			
	*gltA*	SFGgltA71F	GTTCAGGGTCTTCGTGCATTTC	1156	98 °C for 1 min, 45 cycles of 98 °C for 10 s, 60 °C for 20 s, 72 °C for 1min, hold at 72 °C for 2 min	[43]
		SFGgltA1226R	AAAGCAAGTATCGGTGAGGATG			
Colorado tick fever virus	Viral protein 12	CTFV-VP12-Fw	TTCGGCAGTACGCAATGT	114	95 °C for 10 min, 40 cycles of 95 °C for 11 s, 60 °C for 30 s, hold at 72 °C for 2 min	Designed ^b^
		CTFV-VP12-Rv	AAATCGGCGGGAGAATAA			

^a^ PCR and Sanger sequencing with the following primers were used only for sample H17 to obtain a longer amplicon for improved phylogenetic resolution. ^b^ Primer design provided by Dr. Saravanan Thangamani, Director, SUNY Center for Environmental Health and Medicine/Vector Biology Laboratories, NY, USA.

## Data Availability

The original data presented in the study are openly available in the International Nucleotide Sequence Database under accession numbers PZ439541-PZ439543.

## Supplementary Materials

The following supporting information can be downloaded at: https://www.mdpi.com/article/10.3390/pathogens15080855/s1, Table S1: GenBank accession numbers used in the *Rickettsia* phylogenetic analysis; Figure S1: Phylogenetic tree based on the partial sequence of the COI gene of *D. similis* samples collected in this study. Sequences generated in this study are shown in Bold text, with the sample number and the corresponding collection counties: S, Siskiyou; H, Humboldt. Reference sequences of previously reported *D. similis* and *D. variabilis* were obtained from GenBank with the accession numbers. Sequences from *D. andersoni* were used to root the tree. The tree was constructed using the maximum likelihood method and the Tamura 3-parameter model (1992) + G in MEGA X with bootstrap values (1000 replicates) shown at the nodes; Figure S2: Multiple sequence alignment of CTFV VP12 gene sequences. Alignment of representative CTFV-like sequence obtained in this study (S18) and reference sequences retrieved from GenBank, including CTFV strain Florio (MZ484433), isolate CTFV-S6-14-03 (AF343060), R94918b (OQ833598), S6-16-09 (OQ095382), R124791 (MZ484445), Da375 (KY454367), and Da412 (KY454373). A CTFV VP12-like sequence isolate SS-18 (OR839983) is included for comparison. The consensus sequence is shown above the aligned sequences. Alignment was performed using Clustal Omega v. 1.2.4 on SnapGene v. 8.2.2.
