# Supplementary material for: Molecular Surveillance Reveals Diverse Tick-Borne Pathogens in Northern California
Source: Pathogens. 2026 Aug 17;15(8):855. doi: 10.3390/pathogens15080855 (PMC13516315; doi:10.3390/pathogens15080855)
Supplement: Supplementary file 1 [file pathogens-15-00855-s001.zip › pathogens-4452602-supplementary.pdf]

Table S1: GenBank accession numbers used in the *Rickettsia* phylogenetic analysis.

Species	*ompA*	*ompB*	*gltA*
R. aeschlimannii	U83446	HM050278	HM050276
R. africae	CP001612	CP001612	CP001612
R. akari	CP000847	CP000847	CP000847
R. australis	CP003338	CP003338	CP003338
R. canadensis	CP000409	CP000409	CP000409
R. conorii	AE006914	AE006914	AE006914
R.conorii subsp. *heilongjiangensis*	CP002912	CP002912	CP002912
R. conorii subsp. *raoultii*	CP010969	CP010969	CP010969
R. felis	CP000053	CP000053	CP000053
R. honei	AF018076	AF123711	U59726
R. japonica	AP011533	AP011533	AP011533
R. lanei	JN990595	CP172233	CP172233
R. massiliae	CP000683	CP000683	CP000683
R. monacensis str. IrRMunich	LN794217	LN794217	LN794217
R. monacensis str. Humboldt	LAOP01000001	KJW03404	LAOP01000001
R. montanensis	CP003340	CP003340	CP003340
R. parkeri	CP003341	CP003341	CP003341
R. peacockii	CP001227	CP001227	CP001227
R. philipii	CP003308	CP003308	CP003308
R. rhipicephali	CP013133	CP003342	CP003342
R. rickettsii	CP003311	CP003311	CP003311

R. sibirica subsp. *mongolitimonae*	U83439	DQ097083	AABW01000001
R. slovaca	CP003375	CP003375	CP003375
R. tamurae subsp. *buchneri*	JFKF01000169	JFKF01000150	JFKF01000076

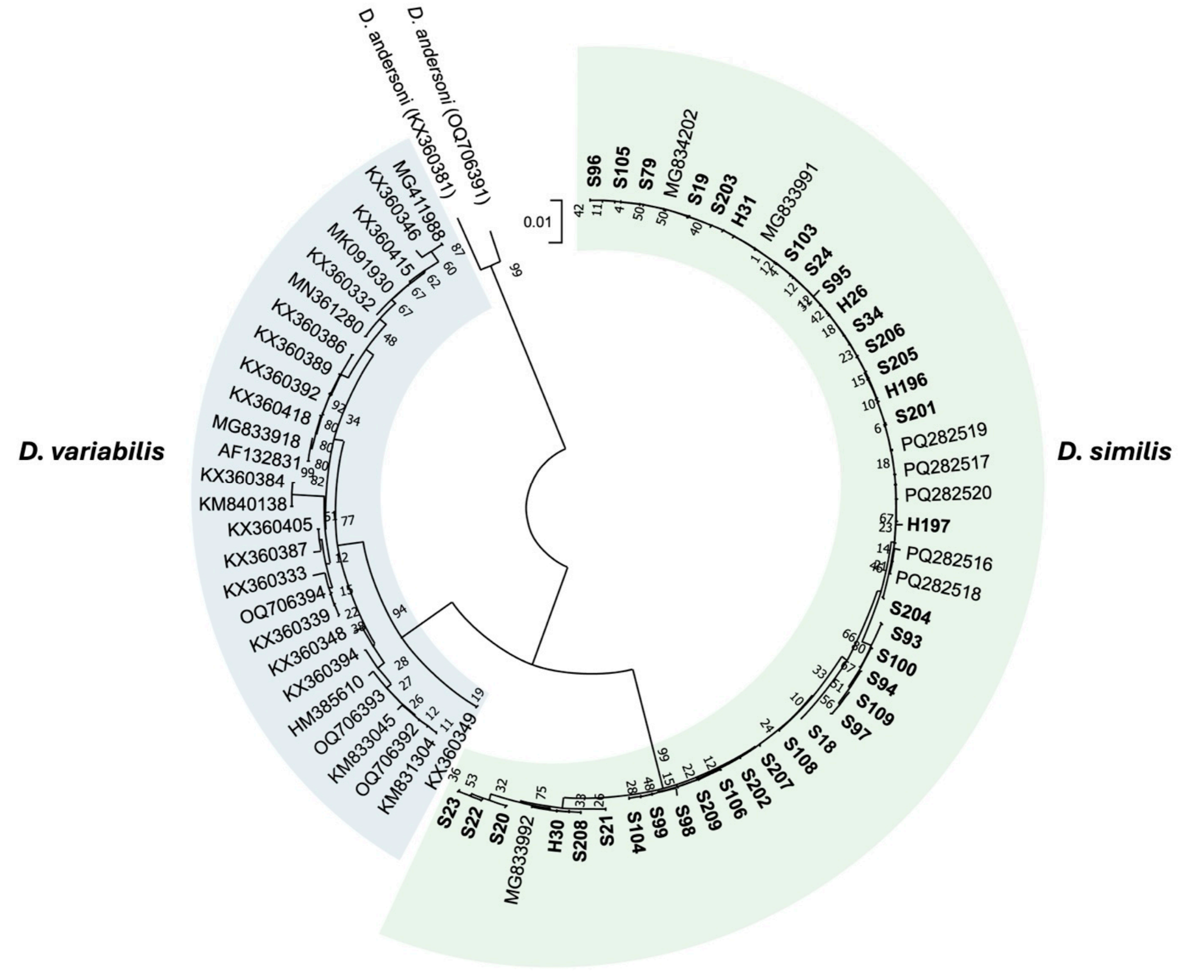

Figure S1: Phylogenetic tree based on the partial sequence of the COI gene of *D. similis* samples collected in this study. Sequences generated in this study are shown in Bold text, with the sample number and the corresponding collection counties: S, Siskiyou; H, Humboldt. Reference sequences of previously reported *D. similis* and *D. variabilis* were obtained from GenBank with the accession numbers. Sequences from *D. andersoni* were used to root the tree. The tree was constructed using the maximum likelihood method and the Tamura 3-parameter model (1992) + G in MEGA X with bootstrap values (1000 replicates) shown at the nodes.

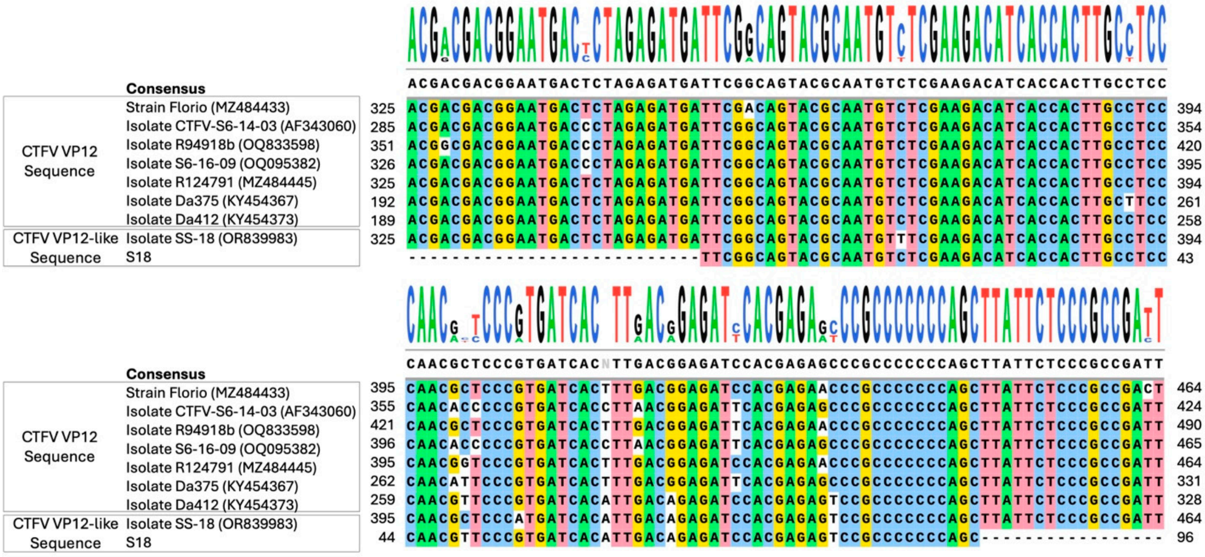

Figure S2: Multiple sequence alignment of CTFV VP12 gene sequences. Alignment of representative CTFV-like sequence obtained in this study (S18) and reference sequences retrieved from GenBank, including CTFV strain Florio (MZ484433), isolate CTFV-S6-14-03 (AF343060), R94918b (OQ833598), S6-16-09 (OQ095382), R124791 (MZ484445), Da375 (KY454367), and Da412 (KY454373). A CTFV VP12-like sequence isolate SS-18 (OR839983) is included for comparison. The consensus sequence is shown above the aligned sequences. Alignment was performed using Clustal Omega v. 1.2.4 on SnapGene v. 8.2.2.
